# Intercensal and Postcensal Estimation of Population Size for Small Geographic Areas in the United States

**DOI:** 10.23889/ijpds.v5i1.1160

**Published:** 2020-08-13

**Authors:** Y Wang, X Zhang, H Lu, KA Matthews, KJ Greenlund

**Affiliations:** 1 Centers for Disease Control and Prevention, 4770 Buford Highway, NE Atlanta, GA 30341; 2 Economic Research Service, United States Department of Agriculture, Washington, DC, USA; 3 Bureau of Labor Statistics, United States Department of Labor, Washington, DC, USA

**Keywords:** Interpolation, extrapolation, population estimation, decennial census

## Abstract

**Introduction:**

Population estimation techniques are often used to provide updated data for a current year. However, estimates for small geographic units, such as census tracts in the United States, are typically not available. Yet there are growing demands from local policy making, program planning and evaluation practitioners for such data because small area population estimates are more useful than those for larger geographic areas.

**Objectives:**

To estimate the population sizes at the census block level by subgroups (age, sex, and race/ethnicity) so that the population data can be aggregated up to any target small geographic areas.

**Methods:**

We estimated the population sizes by subgroups at the census block level using an intercensal approach for years between 2000 and 2010 and a postcensal approach for the years following the 2010 decennial census (2011-2017). Then we aggregated the data to the county level (intercensal approach) and incorporated place level (postcensal approach) and compared our estimates to corresponding US Census Bureau (the Census) estimates.

**Results:**

Overall, our intercensal estimates were close to the Census’ population estimates at the county level for the years 2000-2010; yet there were substantive errors in counties where population sizes experienced sudden changes. Our postcensal estimates were also close to the Census’ population estimates at the incorporated place level for years closer to the 2010 decennial census.

**Conclusion:**

The approaches presented here can be used to estimate population sizes for any small geographic areas based on census blocks. The advantages and disadvantages of their application in public health practice should be considered.

## Introduction

Population size plays a critical role in both public and private sectors for various purposes, such as allocation of resources and funds, environmental planning, public service delivery, estimation of disease rates, and measurement of the association between environmental exposure and health outcomes. Census data is a traditional and reliable source for population size at multiple geographic areas in the United States and other countries. However, counting population every tenth or fifth year fails to account for population size and unit of geography changes from year to year. Additionally, the needs for small area population estimates and their demographic characteristics that can be aggregated to all higher geographies continues to grow. These estimates can be used as denominators for rates, controls for demographic surveys, evidence to guide administrative planning, small area estimation, and many other applications. To fill these gaps, population estimation techniques have been developed and used in the world for the intervening years (see reviews [1, 2]).

The cohort component method [3] is a standard demographic method that is widely used in many countries. It accounts for births, deaths, and net migration. The Census Bureau (the Census) in the United States uses this method to produce annual population estimates by subgroups (age, gender, and race/ethnicity) at the national, state, and county-level (n=3,142) based on the most recent census [4]. However, this method is difficult to apply to smaller areas because vital statistics and migration data are not easily available for areas below the county level. Therefore, the Census uses a distributive housing unit method [5, 6] to produce the total estimates for minor civil divisions (e.g., towns and townships) and incorporated places (e.g., cities, boroughs, and villages). However, the method does not provide subgroup specific population estimates directly, and local jurisdictional boundaries do not necessarily align with census boundaries. Some other population estimation approaches for small areas and the demographic characteristics include iterative proportional fitting approach [7, 8], censal-ratio method [9] , statistical techniques that range from simple linear change to complex models [10-12], and approaches utilizing remote sensing and geographic information system(GIS) technologies [13, 14]. Each approach has its own advantages and disadvantages, but most of these approaches are too complex, which may limit their application in practice.

In the present study, we estimated population sizes for small areas by demographic groups (age, sex, and race/ethnicity) as they change over time. Intercensal estimate are calculated using data in which a beginning and end years are reported and postcensal estimates are calculated using data with only a beginning year. However, precensal estimates could also be calculated using this method when using an end year of data. We generated these estimates at the census block-level because it is the smallest geographic unit [15] in the U.S. Census geographic hierarchy [16], which allows us to aggregate up to any small area geographies, such as census tracts, counties, ZIP Code tabulation areas. First, we calculated census block-level intercensal estimates of population size by subgroups at the census block level using 2000 and 2010 census population data. To evaluate the accuracy of these estimates, we aggregated them to the county level by the subgroups so that they could be compared with the Census’ county-level population estimates. Second, we calculated census block-level postcensal estimates of population sizes by subgroups at the block level for years 2011-2017 following the 2010 decennial census. Similarly, we aggregated these estimates to the incorporated place level so that they could be compared with estimates provided by the Census.

## Methods

### Intercensal estimation

One of the challenges encountered in intercensal estimation for small areas is the change in geographic boundaries over time between decennial censuses. For the intercensal period between 2000 and 2010, a block Please in 2000 may have been divided into multiple parts to form several new blocks in 2010 or may have merged with other partial blocks to form a new block in 2010. To account for these changes, we redistributed the 2010 block-level population counts to the 2000 decennial census using the Census 2000 Tabulation Block to 2010 Census Tabulation Block Relationship File [17]). AREALAND_INT and AREALAND_2000 are variables in the Relationship File that indicate intersection of land area shared by the 2000 and 2010 blocks represented by the record and the 2000 land area, respectively. We used a ratio of AREALAND_INT to AREALAND_2000 as a weight to the 2000 population counts for each block part and then summed them to the population for each block. A ratio of population at each block part over population at that block was then applied to the block-level population counts by subgroups (age group in 5-year intervals, sex, and race/ethnicity) in the 2000 decennial census data. Next, we used the two decennial census population counts by subgroups (Pop_2000 and Pop_2010, respectively) as the estimate base and assigned different weights (1- n/10 as weight_2000 and n/10 as weight_2010, n=1, 2, ….9) to each intercensal year based on how close it is to either of the two decennial census. For any intercensal year, we estimated its population by calculating Pop_2000*weight_2000 + Pop_2010*weight_2010. For example, the population in 2001 was estimated as Pop_2000*0.9+Pop_2010*0.1. Finally, we aggregated these block-level population estimates by subgroups to total county-level population estimates and compared them with those provided by the Census [18] for each intercensal year. The commonly used Absolute Percent Error (APE) was used to measure the difference between our estimates and those from the Census. Then we selected the middle year, 2005, to compare the two sets of county-level population estimates by age groups (10-year intervals), sex, and race/ethnicity. Given that the population size in some of the subgroups could be very small or even zero, we used the ratio of the Mean Absolute Error (MAE, calculated as the mean of the absolute error between our estimates and the Census’ estimates), to the mean of the Census’ estimates to compare estimates for this portion of the analysis rather than using APE.

### Postcensal estimation

In this approach, we estimated the block-level population by subgroups for a current year ($N_{bi\_current}$) for the years 2011 to 2017. The Census has both block-level and county-level population data by subgroups for 2010 ($N_{bi_{-}2010}$ and $N_{ci_{\_}2010}$, respectively) based on the April 1, 2010 census counts [19] . We assumed that the proportion of the population by subgroups at the block level within a county in 2010 ($P_{bi}=N_{bi\_2010}/N_{ci\_2010}$) remained the same in subsequent years. For 2011-2017, we applied this proportion to Census postcensal county-level estimates to arrive at block-level population estimates by subgroup ($N_{bi_{\_}current}=P_{bi}*N_{ci\_current}$). Then $N_{bi\_current}$ was aggregated to the incorporated place-level $N_{pi\_current}$ for each year and compared to the Census data. The difference between our postcensal estimates and the Census estimates was measured by APE. Both the county-level population estimates by subgroups and the incorporated place-level estimates were downloaded from the Census’ website [20].

## Results

Table 1 compares the county-level intercensal population estimates generated from our block-based method with those produced by the Census and presents the APEs for 3,143 US counties. Overall, the two sets of the estimates were very close. Relatively higher errors were found in the middle years (farther from either census year) and the highest error at the 90th percentile was 3.6% in 2006. The APEs were especially high for estimates of maximal county population for the years 2006 (192.4%) and 2007 (91.8%). Some of these discrepancies could be explained by the sudden changes in population size due to natural disasters, such as Hurricane Katrina. Figure 1 illustrates that we underestimated the population size before 2005 (APE in 2005 = 27.7%) and overestimated it after 2005 (APE in 2006 = 192.4%) for St. Bernard Parish, Louisiana because it was damaged by Katrina; whereas in Lincoln Parish, Louisiana, which was not affected by Hurricane Katrina, our estimates were very close to the estimates published by the Census. For the population size by subgroups, we selected the year 2005 to compare our intercensal estimates with the Census’ data (Table 2). Higher errors were associated with subgroups of small population size, such as American Indian/Alaska Natives in the ≥60 years age groups, and Native Hawaiian/Pacific Islanders and two or more races in all age groups. The distribution of errors were quite similar between males and females, though.

**Figure 1: Comparison between block-based intercensal population estimates and the Census’ estimates for St. Bernard Parish, Louisiana (left) and Lincoln Parish, Louisiana (right) between 2000 and 2010.**
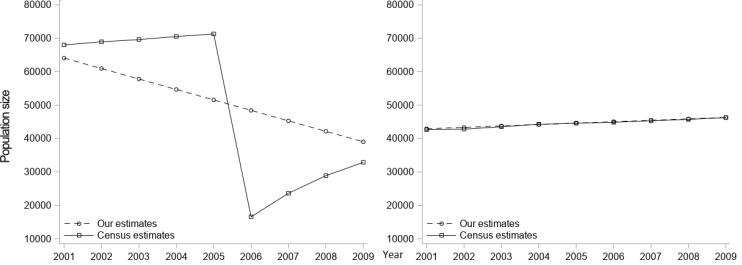


**Table 1: Distribution of block-based intercensal population estimates, Census intercensal population estimates, and the APEs among 3,143 US counties:** Abbreviation: APE, absolute percent error.

		Min	25^th^	50^th^	75^th^	90^th^	Max
2001						
	Block-based estimates	68	11,135	24,808	62,332	178,137	9,549,064
	Census estimates	66	11,148	24,690	62,083	176,496	9,626,034
	APE	0	0.3	0.6	1.2	1.9	12.1
2002						
	Block-based estimates	70	11,176	24,960	62,729	178,697	9,579,013
	Census estimates	75	11,155	24,962	62,547	178,464	9,705,913
	APE	0	0.4	0.8	1.6	2.6	21.5
2003						
	Block-based estimates	71	11,191	25,079	62,873	180,096	9,608,962
	Census estimates	72	11,120	25,060	62,947	181,976	9,767,145
	APE	0	0.5	1.0	1.8	3.2	16.9
2004						
	Block-based estimates	73	11,171	25,172	63,700	181,535	9,638,911
	Census estimates	55	11,153	25,154	63,699	184,939	9,793,263
	APE	0	0.5	1.1	2.0	3.4	32.7
2005						
	Block-based estimates	74	11,226	25,147	64,505	185,538	9,668,860
	Census estimates	70	11,142	25,299	64,718	186,543	9,786,373
	APE	0	0.5	1.1	2.1	3.5	27.7
2006						
	Block-based estimates	76	11,173	25,260	64,745	187,973	9,698,809
	Census estimates	75	11,138	25,463	65,402	189,682	9,737,955
	APE	0	0.5	1.1	2.1	3.6	192.4
2007						
	Block-based estimates	77	11,132	25,388	65,543	190,010	9,728,758
	Census estimates	79	11,061	25,550	65,740	192,837	9,700,359
	APE	0	0.5	1.0	2.0	3.3	91.8
2008						
	Block-based estimates	79	11,121	25,566	65,554	194,010	9,758,707
	Census estimates	61	11,084	25,699	66,086	195,094	9,735,147
	APE	0	0.4	0.9	1.6	2.7	46.0
2009						
	Block-based estimates	80	11,107	25,725	66,228	195,475	9,788,656
	Census estimates	77	11,101	25,790	66,518	197,192	9,787,400
	APE	0	0.2	0.5	1.0	1.6	18.7

### Main results

Considering individual-level household income quintile and neighbourhood-level material deprivation quintile as separate exposures, in age-, sex-, and cycle-adjusted models, risk of avoidable hospitalization increased in a graded manner across both income quintiles and deprivation quintiles (Model 1) (Table 2). Adjustment for demographic variables slightly increased income effect sizes but had no effect on deprivation effect sizes (Model 2). Additional adjustment for other socioeconomic variables attenuated effect sizes, particularly for income quintiles 1 and 2 and deprivation quintiles 3-5 (Model 3). Here, individuals in the lowest income quintile and those living in the most deprived neighbourhoods were more than twice as likely to experience an avoidable hospitalization relative to those in the highest income quintile and living in the least deprived neighbourhoods, respectively. Final adjustment for behavioural variables further attenuated the effects of household income and material deprivation on risk of avoidable hospitalization (Income: RR 1.82 (1.56-2.13) Deprivation: RR 1.67 (1.44-1.95)) (Model 4). When both individual-level household income quintile and neighbourhood-level material deprivation quintile were entered in the model together, a similar pattern was observed with more attenuated effect sizes relative to the single exposure models.

**Table 2: Ratio of MAE* to mean Census’ estimates (%) by age group, sex, and race/ethnicity among 3,143 counties for 2005:** Abbreviations: AIAN, American Indian/Alaska Native; NHPI, Native Hawaiian/Pacific Islander; MAE, mean absolute error. * MAE is calculated as the mean of the absolute error between block-based estimates and the Census’ estimates.

	Non-Hispanic						Hispanic
		
Age group (years)	White	Black	AIAN	Asian	NHPI	Two or more
Male						
	0-9	3.2	5.9	9.7	5.3	10.2	11.9	3.7
	10-19	3.8	6.6	8.2	4.3	9.8	14.5	4.0
	20-29	3.4	4.0	7.9	5.6	10.3	25.7	3.9
	30-39	4.8	5.4	8.5	7.0	9.0	38.6	4.0
	40-49	5.5	6.6	9.2	5.0	12.6	26.6	3.6
	50-59	4.1	3.8	8.4	5.4	10.4	26.5	3.1
	60-69	5.9	7.6	10.7	5.8	13.1	34.5	7.5
	70-79	3.0	4.5	14.5	5.1	16.2	37.5	3.8
	80+	3.5	6.9	19.4	5.9	23.9	44.1	6.0
Female						
	0-9	3.2	5.9	10.0	5.5	10.4	11.8	3.7
	10-19	3.7	6.5	8.3	4.2	10.3	14.5	3.7
	20-29	3.3	3.6	7.7	6.0	9.8	20.7	3.1
	30-39	4.9	4.8	8.4	5.7	9.4	31.7	3.6
	40-49	5.6	6.6	9.5	4.4	11.8	20.8	3.7
	50-59	3.9	3.8	8.4	5.9	10.8	22.2	3.0
	60-69	5.8	7.3	10.5	8.1	12.2	34.1	6.9
	70-79	3.3	3.8	12.4	5.1	14.6	35.4	3.1
	80+	2.9	5.3	14.7	5.0	18.2	36.5	4.6

Table 3 presents the distributions of our census block-based postcensal population estimates, the Census’s postcensal population estimates, and the APEs by incorporated place for 2011-2017. There were 19, 471 incorporated places in total, however 18 were excluded because they were formed after the 2010 Decennial Census. Generally, the distributions of the two sets of estimates were close in magnitude and the errors were in a reasonable range. It also shows that the error levels increase with each passing year, which indicates that our assumption that the percentage of block-level/county-level population size ($P_{bi}$) calculated in 2010 remains the same becomes less valid as we get further from 2010. For the large discrepancies in 2016 and 2017 shown in Table 3, we found that they could be due to the different 2010 estimate base that we used. Our postcensal estimation was based on the 2010 census counts, while the Census adjusted this base to reflect changes to the 2010 census population from the Count Question Resolution program, legal boundary and other geographic updates, and edits to the race categories [1]. Table 4 shows how this difference in 2010 values can have a profound effect on postcensal estimates for a few select incorporated places.

**Table 3: Distribution of block-based intercensal population estimates, Census intercensal population estimates, and the APEs among 3,143 US counties:** Abbreviation: APE, absolute percent error.

		Min	25th	50th	75th	90th	Max
2001						
	Block-based estimates	1	368	1,147	4,591	17,553	8,292,686
	The Census’ estimates	1	369	1,149	4,619	17,766	8,292,688
	APE	0	0.3	0.6	1.3	2.5	323.8
2012						
	Block-based estimates	1	367	1,144	4,604	17,722	8,383,502
	The Census’ estimates	1	367	1,149	4,621	17,852	8,333,504
	APE	0	0.4	0.9	1.8	3.5	324.6
2013						
	Block-based estimates	1	366	1,148	4,611	17,846	8,458,640
	The Census’ estimates	1	367	1,148	4,634	17,889	8,458,642
	APE	0	0.5	1.2	2.4	4.5	322.4
2014						
	Block-based estimates	1	366	1,145	4,621	17,887	8,521,132
	The Census’ estimates	1	366	1,145	4,655	18,026	8,521,135
	APE	0	0.7	1.5	3.0	5.6	323.8
2015						
	Block-based estimates	1	365	1,146	4,635	17,997	8,582,455
	The Census’ estimates	1	365	1,145	4,681	18,181	8,582,459
	APE	0	0.8	1.8	3.6	6.6	322.3
2016						
	Block-based estimates	1	365	1,146	4,670	18,053	8,615,419
	The Census’ estimates	1	365	1,145	4,715	18,297	8,615,426
	APE	0	0.9	2.1	4.2	7.7	323.3
2017						
	Block-based estimates	1	365	1,148	4,699	18,155	8,622,690
	The Census’ estimates	1	364	1,150	4,713	18,413	8,622,698
	APE	0	1.1	2.4	4.8	8.8	318.9

**Table 4: Ratio of MAE* to mean Census’ estimates (%) by age group, sex, and race/ethnicity among 3,143 counties for 2005:** Abbreviation: APE, absolute percent error.

	April 1, 2010 Estimates		The Census Bureau’s postcensal estimates		Block-based postcensal estimates		APE	
	Census	base	2011	2017	2011	2017	2011	2017
Vamado village, Louisiana	1,461	340	340	336	1,441	1,408	323.8	319.0
Morgan city, Georgia	240	1861	1,858	1,849	240	232	87.1	87.5
Bristol Village, Wisconsin	2,584	4,876	4,882	5,034	2,581	2,547	47.1	49.4
Pelzer town, South Carolina	89	1,291	1,299	1,373	90	95	93.1	93.1

## Discussion

In the present study, we used intercensal approaches to estimate the population sizes of subgroups at the block level for years between two decennial census years (2000 and 2010) and postcensal approaches for the years 2010-2017. Both approaches presented here were conducted at the block level to allow aggregation to any target small areas by subgroups.

To estimate the population sizes for small areas with demographic characteristics, one would not only consider the estimation errors but also need to take into account constraints of data sources, approach assumptions, complexity, and cost. Compared with most other methods in the current literature that we mentioned before, the methods outlined in this study have some notable advantages. First, decennial Census data are publicly available for all census blocks across the United States which provides local jurisdictions with a reliable source of population data source. Second, the assumptions are relatively straightforward making these methods relatively easy to implement. Third, these methods produce block-level population estimates, which provides more flexibility in generating population estimates for any upper geographic units or locally customized geographic areas. This advantage allows local health departments to produce population estimates for calculating disease rates, mapping disease burdens, or perform other disease surveillance activities for any geographic unit within their jurisdiction.

Some precautions should be noted when utilizing the present procedures to estimate population size for small areas. First, our intercensal estimation assumed that changes to the county-level population sizes are linear between the two decennial census years, but sudden changes in population sizes may be caused by natural or human-made disasters, introduction of new residential subdivisions, and gentrification of existing domiciles. Relatedly, any changes to county-level population sizes are uniformly distributed across the county, which will produce overestimates in some blocks within a county and underestimates in other areas, depending on which areas within the county experienced these sudden population changes. Second, the accuracy of estimation was affected when some subgroup population sizes were too small, such as Native Hawaiian/Pacific Islander population. This problem could be addressed in future research by combining population groups, or combining census blocks, or combining race data from other data sources, such as American Community Survey. Third, when calculating the percentages in both procedures, the population size for a certain subgroup could be zero in the decennial census 2010 but non-zero in the following years. In such a situation, we were not able to obtain percentages. Finally, as our postcensal estimation relied solely on the demographic pattern of the 2010 decennial census, the results may be more useful for years close to the 2010 decennial census than the distant years.

## Conclusion

In this study, we estimated intercensal and postcensal subgroup population sizes at the block level. Local jurisdictions can use this method to calculate estimates that can be easily aggregated to any target small areas. The method itself can be generalized to apply to a wide variety of applications, such as calculating vital rates and small area estimation.

## Statement on Conflicts of Interest

The findings and conclusions in this report are those of the authors and do not necessarily represent the official position of the Centers for Disease Control and Prevention, Economic Research Service, United States Department of Agriculture and Bureau of Labor Statistics, United States Department of Labor. The authors declare no conflicts of interest.

## Ethics Statement

All the data in this study are publicly available thus ethics review and approval were exempted.

## Abbreviations

APE	absolute percent error
CDC	Centers for Disease Control and Prevention
The Census	the U.S. Census Bureau
MAE	mean absolute error
